# *In vitro* and *in vivo* effects of a recombinant anti-PSMA immunotoxin in combination with docetaxel against prostate cancer

**DOI:** 10.18632/oncotarget.8001

**Published:** 2016-03-09

**Authors:** Marta Michalska, Susanne Schultze-Seemann, Lioudmila Bogatyreva, Dieter Hauschke, Ulrich Wetterauer, Philipp Wolf

**Affiliations:** ^1^ Department of Urology, Medical Center, University of Freiburg, Freiburg, Germany; ^2^ Institute for Medical Biometry and Statistics, Medical Center, University of Freiburg, Freiburg, Germany

**Keywords:** prostate cancer, PSMA, immunotoxin, docetaxel, chemotherapy

## Abstract

Docetaxel (DOC) is used for the first-line treatment of castration resistant prostate cancer (CPRC). However, the therapeutic effects are limited, only about one half of patients respond to the therapy and severe side effects possibly lead to discontinuation of treatment. Therefore, actual research is focused on the development of new DOC-based combination treatments.

In this study we investigated the antitumor effects of a recombinant immunotoxin targeting the prostate specific membrane antigen (PSMA) in combination with DOC *in vitro* and *in vivo*. The immunotoxin consists of an anti-PSMA single chain antibody fragment (scFv) as binding and a truncated form of *Pseudomonas aeruginosa* Exotoxin A (PE40) as toxin domain. The immunotoxin induced apoptosis and specifically reduced the viability of androgen-dependent LNCaP and androgen-independent C4-2 prostate cancer cells. A synergistic cytotoxic activity was observed in combination with DOC with IC_50_ values in the low picomolar or even femtomolar range. Moreover, combination treatment resulted in an enhanced antitumor activity in a C4-2 SCID mouse xenograft model. This highlights the immunotoxin as a promising therapeutic agent for a future DOC-based combination therapy of CPRC.

## INTRODUCTION

Prostate cancer remains the most common cancer and the second most leading cause of cancer deaths in industrial countries [1]. Although incidence and death rate of prostate cancer are on the decrease, there is currently no curative treatment available for advanced stages.

As prostate tumor growth in general is androgen-dependent, androgen deprivation therapy (ADT) is considered as the standard therapy for men with *de novo* or recurrent metastatic disease [2]. ADT prolongs overall survival and commonly leads to an initial clinical response [2]. However, several months after beginning ADT leads to an aggressive, androgen-independent grow of tumors cells and virtually all patients show tumor progression [3]. This stage is defined as castration resistant prostate cancer (CRPC). In 2004, the FDA approved the cytotoxic antimicrotuble agent Docetaxel (DOC, Taxotere®, Aventis Pharmaceuticals, Inc.) for use in combination with prednisone for the treatment of CRPC. Unfortunately, overall survival rate is only slightly increased by 2.5 months with this treatment option and only about half of men generally respond to this therapy. Moreover, DOC can provoke severe side effects and treatment has possibly to be discontinued because of toxicity or disease progression [4, 5]. Therefore, actual research is focused on new DOC-based combinatorial treatments to improve therapeutic efficacy and to reduce the side effects. For example, in preclinical studies the combination of the apoptosis inducing protein GLIPR1-ΔTM, the vasopressin analogue desmopressin, the synthetic somatostatin analogue octreotide or the inhibitor rapamycin led to enhanced cytotoxic effects of DOC [6–9]. In a mouse model with patient-derived tissue xenografts, the combination of Aneustat™ (OMN54), a multifunctional botanical anti-cancer drug candidate, provoked enhanced antitumor activity with DOC [10]. Moreover, in a first clinical trial low-dose DOC in combination with dexamethasone led to a reduced hematological toxicity in CPRC patients compared to the DOC standard therapy [11].

In the present study, we describe the preclinical evaluation of a recombinant immunotoxin targeting the prostate specific membrane antigen (PSMA) in combination with DOC. PSMA is a transmembrane protein, which is highly restricted to the surface of prostate cancer cells [12]. It is present on all tumor stages without secretion into the extracellular space and is able to internalize after antibody binding [13–15]. These characteristics as well as its enhanced expression in androgen-independent and metastatic disease make it an ideal candidate for the targeted treatment of CPRC [16–18].

For the construction of our immunotoxin, the scFv D7, generated from our anti-PSMA monoclonal antibody (mAb) 3/F11, was used as binding domain [12, 19, 20]. The truncated form of *Pseudomonas* Exotoxin A (PE40) from the bacterium *Pseudomonas aeruginosa* was chosen as the toxin domain. PE40 consists of the transmembrane domain II to permeate cellular membranes, and the domains Ib and III, which have ADP-ribosylation activity. *Pseudomonas* exotoxin A is able to specifically ADP-ribosylate the eukaryotic elongation factor 2 (eEF-2) on the ribosomes. ADP-ribosylation of eEF-2 leads to an inhibition of protein biosynthesis and to apoptosis of the target cell [21].

## RESULTS

### Cloning, expression and purification of the anti-PSMA immunotoxin D7(VL-VH)-PE40

**Figure 1:**
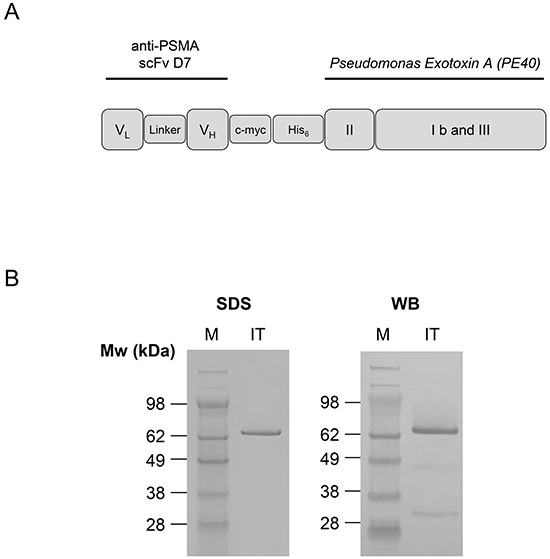
Cloning, expression and purification of the anti-PSMA immunotoxin D7(VL-VH)-PE40 **A.** Schematic representation of the recombinant anti-PSMA immunotoxin D7(VL-VH)-PE40 consisting of the anti-PSMA scFv D7 linked to the cytotoxic part of *Pseudomonas* Exotoxin A (PE40). PE40 consists of the transmembrane domain II and the domains Ib and III with ADP-ribosyltransferase activity. A human c-myc-tag as well as a hexahistidine tag were inserted for detection and purification of the immunotoxin, respectively. **B.** SDS-PAGE and Western Blot of the purified anti-PSMA immunotoxin D7(VL-VH)-PE40. Abbreviations: c-myc, human c-myc tag; His_6_, hexahistidine tag; IT, immunotoxin D7(VL-VH)-PE40; linker, GGGS; PSMA, prostate specific membrane antigen; scFv, single chain; V_H_, variable domain of the antibody heavy chain; V_L_, variable domain of the antibody light chain; WB, Western-Blot.

The immunotoxin, called D7(VL-VH)-PE40, was recombinantly made by N-teminally cloning the anti-PSMA scFv D7 in a VL-VH orientation to the PE40 domain into the expression vector pHOG21 (Figure 1A). The correctness of the DNA sequence and the integration into the genome of *E.coli* XL1-blue bacteria clones were confirmed by Sanger sequencing (GATC, Konstanz, Germany). D7(VL-VH)-PE40 was periplasmatically expressed to ensure proper folding and subsequently purified by immobilized metal ion affinity chromatography (IMAC). An amount of about 1.2 mg immunotoxin per liter bacteria culture was yielded. The high purity of the preparations was verified by SDS-PAGE. Western-Blot analysis confirmed the expression of the 70 kDa protein (Figure 1B).

### Binding and internalization of D7(VL-VH)-PE40 into PSMA expressing prostate cancer cells

**Figure 2:**
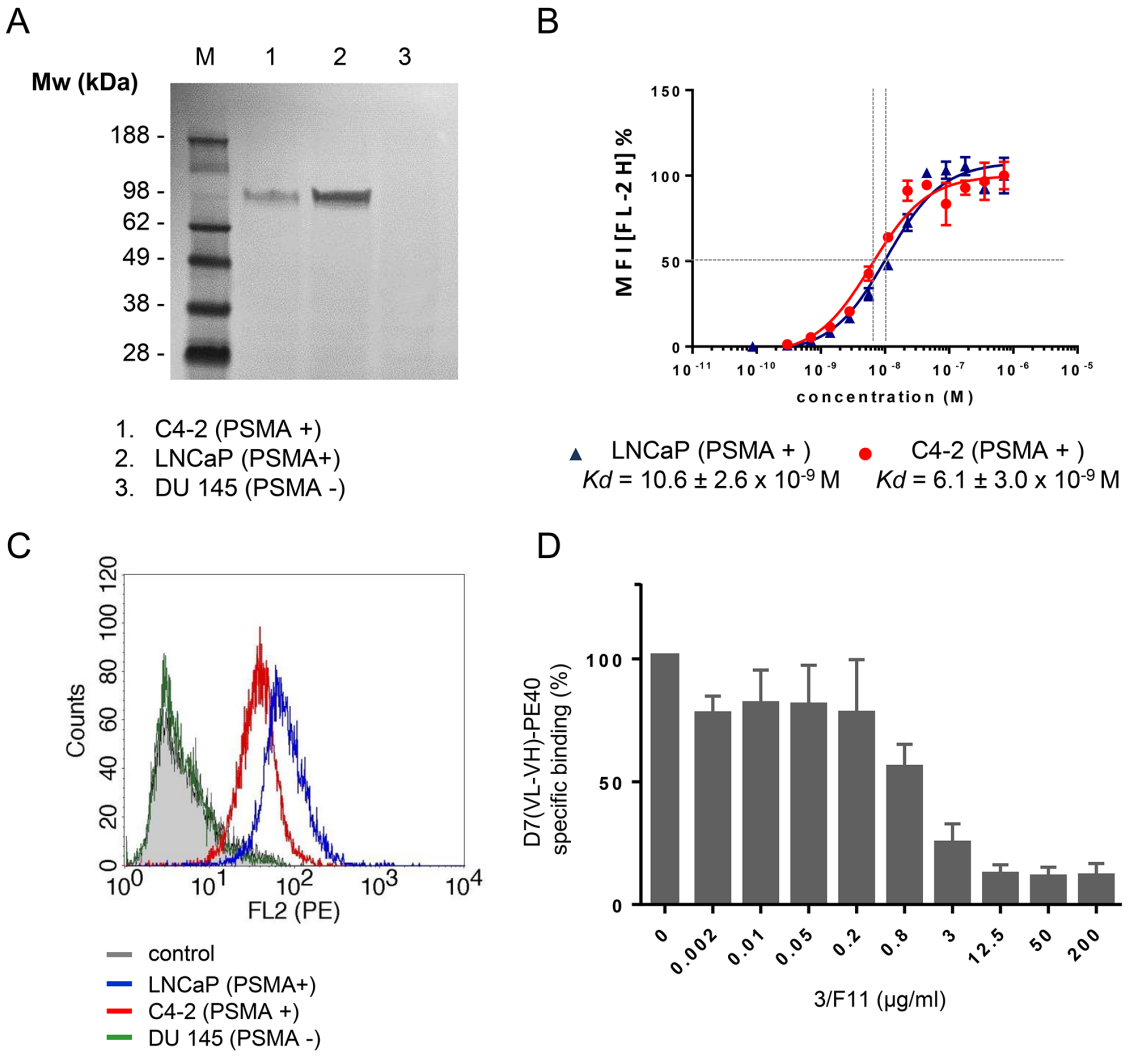
Binding of D7(VL-VH)-PE40 to PSMA positive prostate cancer cells **A.** Western Blot of PSMA expression in C4-2, LNCaP, and DU 145 cells (control). **B.** Binding of the immunotoxin was tested on LNCaP (-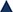-) and C4-2 (-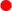-) cells by flow cytometry. Data of three independent experiments are presented as mean +/− SD. **C.** Cell binding of the immunotoxin at saturating concentration. **D.** Inhibition of D7(VL-VH)-PE40 binding to C4-2 cells by parental mAb 3/F11. Cells were treated with various concentrations of 3/F11 and further incubated with 5 μg/ml immunotoxin. The immunotoxin was then labeled with rat anti-human c-myc mAb and goat anti-rat IgG(H+L)-R-PE. Data are shown as mean +/− SD. M, standard protein marker.

PSMA expression of the LNCaP and C4-2 cells was verified by Western-Blotting. DU 145 control cells were shown to be PSMA negative (Figure 2A).

Binding of the anti-PSMA immunotoxin D7(VL-VH)-PE40 to LNCaP and C4-2 cells was demonstrated by flow cytometry. The apparent binding affinities (*Kd*) were determined by calculating the concentration of immunotoxin that produced half-maximal specific binding. The *Kd* values of D7(VL-VH)-PE40 were 10.6 ± 2.6 × 10^−9^ M on LNCaP and 6.1 ± 3.0 × 10^−9^ M on C4-2 cells, respectively (Figure 2B). No binding was seen to PSMA negative DU 145 control cells (Figure 2C). Cell binding was blocked by preincubation with increasing concentrations of 3/F11, the parental anti-PSMA mAb of the scFv D7 (Figure 2D). This confirms that the immunotoxin retained the specific binding to the same extracellular PSMA epitope.

**Figure 3:**
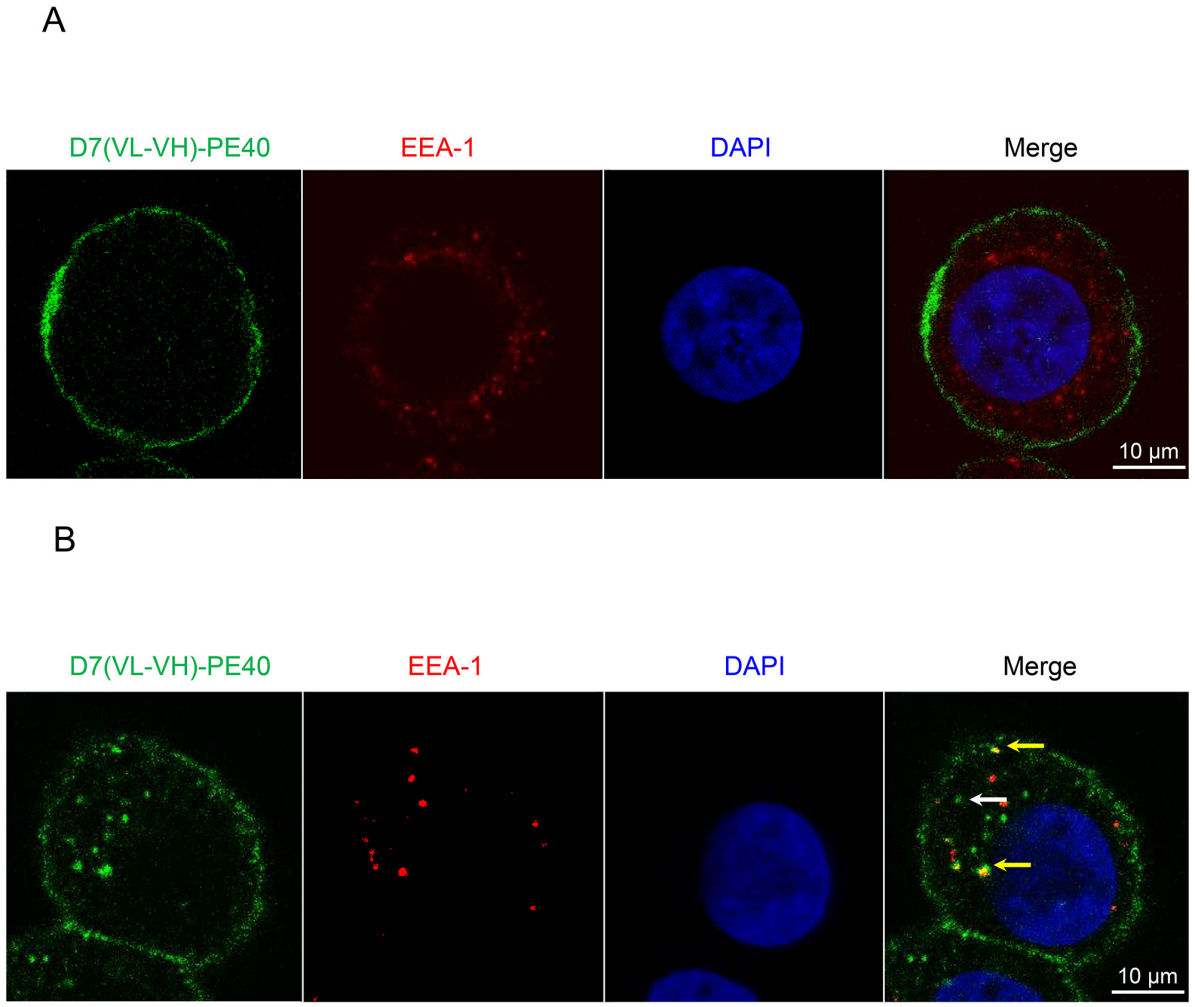
Binding and internalization of D7(VL-VH)-PE40 into PSMA positive cells C4-2 cells were incubated with immunotoxin at **A.** 4°C or **B.** 37°C, 5% CO_2_, for 4 h. Then the immunotoxin was stained with mouse anti-human c-myc mAb and goat anti-mouse-IgG-AF488 (green). Early endosomes were labeled with rabbit anti-human early endosome antigen 1 (EEA-1) and anti-rabbit-IgG-DyLight®650 (red). Nuclei were stained with DAPI (blue). Images were taken by Confocal Laser Scanning Microscopy. D7(VL-VH)-PE40 bound at the cell surface at both temperatures. At 37°C the immunotoxin also localized within endosomes (yellow merge, yellow arrows) as well as in other compartments inside the cells (white arrow).

The immunotoxin needs to be internalized via endocytosis, so that it can reach the cytosol to induce its cytotoxic effect [21]. To examine the internalization and cellular localization within prostate cancer cells, colocalization of the immunotoxin with endosomes was investigated by Confocal Laser Scanning Microscopy. As shown in Figure 3A, there was a strong binding of the immunotoxin to the cell surface at 4^o^ C, a temperature, which prevents metabolic activity including internalization of the PSMA/immunotoxin complex. Under physiological conditions (37°C, humidified atmosphere of 5% CO_2_) internalized immunotoxin was observed inside endosomes (yellow merge) and in other compartments inside the cells (Figure 3B).

### Cytotoxicity of D7(VL-VH)-PE40 in combination with DOC in prostate cancer cells

**Figure 4:**
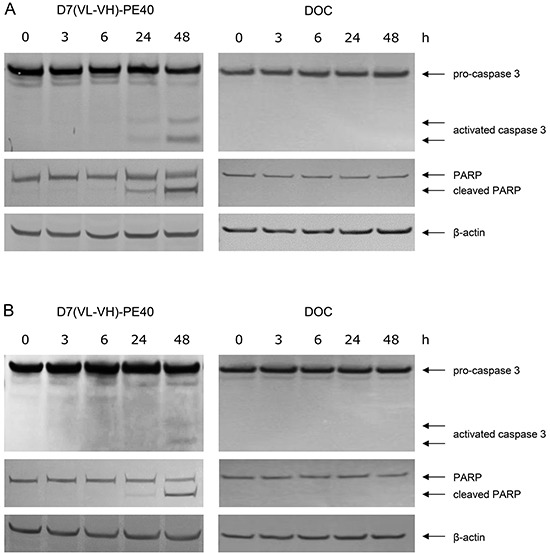
Induction of apoptosis by D7(VL-VH)-PE40 and DOC in prostate cancer cells Apoptotic action of 1×10^−9^ M D7(VL-VH)-PE40 and a subtoxic concentration of 4×10^−9^ M DOC was verified by Western-Blotting through caspase 3 activation and poly ADP ribose polymerase (PARP) cleavage. **A.** LNCaP and **B.** C4-2 cells after exposure up to 48 h. β-actin was used as loading control.

**Figure 5:**
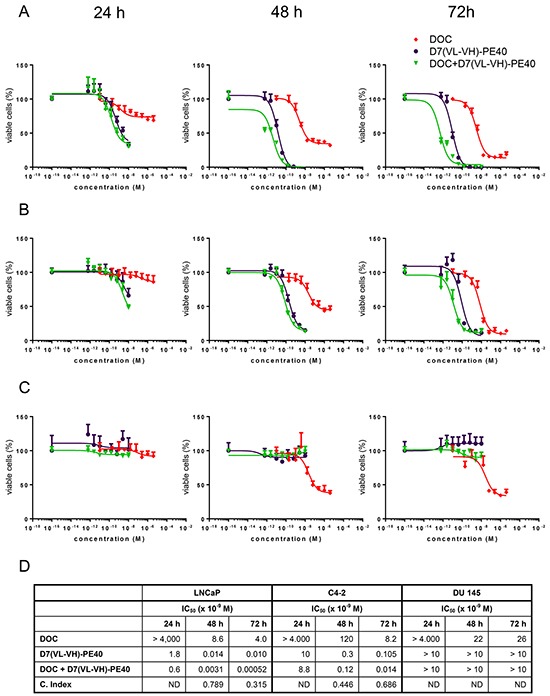
Cytotoxicity of D7(VL-VH)-PE40 in combination with DOC in prostate cancer cells Cytotoxicity in **A.** LNCaP, **B.** C4-2, and **C.** DU 145 cells was investigated by WST-1 cell viability assay. **D.** IC_50_ and Combination Index (C. Index) values of DOC and D7(VL-VH)-PE40. **C.** Index = D1/D2 + Dx1/Dx2 + (D1*D2) / Dx1*Dx2 with: D1 = concentration of D7(VL-VH)-PE40, which leads to a 50% inhibition of cell viability; D2 = concentration of docetaxel, which leads to a 50% inhibition of cell viability; Dx1 and Dx2 = concentrations of both substances, which lead in combination to a 50% inhibition of cell viability. ND = not determinable.

D7(VL-VH)-PE40 was able to induce apoptosis in LNCaP and C4-2 cells after 24–48 h incubation as demonstrated by caspase 3 activation and poly ADP ribose polymerase (PARP) cleavage. No apoptosis was detected after exposure with a low dose of 4.0 × 10^−9^M DOC, which was used in the following combination experiments (Figure 4). The cytotoxicity of the anti-PSMA immunotoxin alone and in combination with DOC was examined by the WST viability assay. On LNCaP cells, DOC alone caused a dose- and time-dependent cell killing with mean IC_50_ values of > 4.0 × 10^−6^ M, 8.6 × 10^−9^ M and 4.0 × 10^−9^ M after 24, 48, and 72 h, respectively (Figure 5A). The immunotoxin D7(VL-VH)-PE40 was about 400- to 2000-fold more cytotoxic than DOC effecting IC_50_ values of 1.8 × 10^−9^ M, 1.4 × 10^−11^ M and 1.0 × 10^−11^ M in the same periods of time. After combination of D7(VL-VH)-PE40 with the subtoxic dose of 4.0 × 10^−9^ M DOC a markedly enhanced cytotoxicity was measured. IC_50_ values of 6.0 × 10^−10^ M, 3.1 × 10^−12^ M and 5.2 × 10^−13^ M were reached after 24, 48, and 72 h, respectively. Combination Indices of 0.789 and 0.315 after 48h and 72 h showed that both substances acted synergistically (Figure 5D).

Compared to LNCaP cells, a lower cytotoxicity was measured on C4-2 cells. With DOC mean IC_50_ values of > 4.0 × 10^−6^ M, 1.2 × 10^−7^ M, and 8.2 × 10^−9^ M were reached after 24, 48 and 72 h. D7(VL-VH)-PE40 was about 80- to 400-fold more cytotoxic than DOC with IC_50_ values of 1.0 × 10^−8^ M, 3.0 × 10^−10^ M and 1.1 × 10^−10^ M (Figure 5B). Combination Indices of 0.446 and 0.686 proved that the combination of immunotoxin plus DOC also had a synergistic effect on C4-2 cells (Figure 5D). DOC evoked IC_50_ values of > 4.0 × 10^−6^ M, 2.2 × 10^−8^ M, and 2.6 × 10^−8^ M after 24, 48 and 72 h, on PSMA-negative DU 145 cells (Figure 5C). No cytotoxicity was measured with D7(VL-VH)-PE40 on this cell line, demonstrating the high specificity of the immunotoxin (Figure 5C).

### Antitumor activity of D7(VL-VH)-PE40 in combination with DOC

We initially tested the toxicity of DOC and D7 (VL-VH)-PE40 in tumor-free SCID-mice. In our studies, a maximal tolerable dose (MTD) of 0.3 mg/kg bw for D7(VL-VH)-PE40 was determined. Animals treated with higher immunotoxin doses showed rough fur and apathy and died within 72 hrs. We presume that these signs of toxicity point to a severe hepatotoxicity, which we and others described in detail in mice treated with PE-based immunotoxins [20, 22, 23]. A MTD of 0.8 mg/kg bw was determined for DOC. Mice treated with higher doses were apathetic and showed paralyses of their extremities. These signs are associated with neuromotor toxicity, which was previously described in DOC treated mice [24]. Combinatorial treatment of mice in the therapeutic scheme mentioned below did not evoke any signs of toxicity.

The antitumor activity of D7(VL-VH)-PE40 and DOC alone or in combination was tested in a C4-2 SCID mouse xenograft model, which simulates the CRPC stage in the clinic.

**Figure 6:**
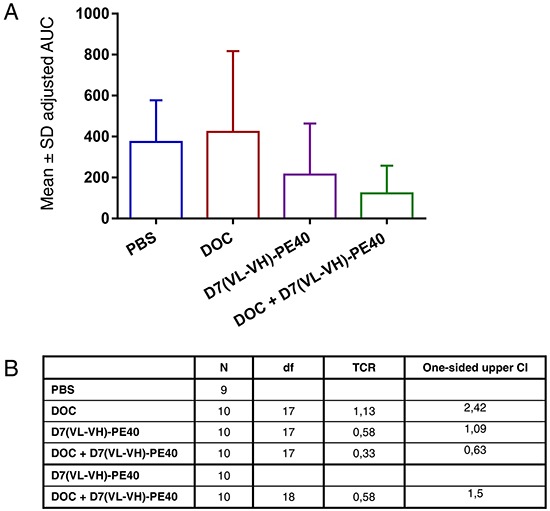
*In vivo* antitumor activity of D7(VL-VH)-PE40 in combination with DOC SCID mice with C4-2 prostate cancer xenografts were treated with DOC, D7(VL-VH)-PE40, or with a combination of both substances. **A.** Error bar adjusted AUC by group. **B.** Pairwise comparisons for treatment to control and treatment to combination treatment groups on time-adjusted AUC. CI, confidence interval for TCR; Df, degrees of freedom; TCR, treatment to control ratio estimator.

Tumor growth inhibition was characterized by the time-adjusted Area-Under-the-Curve (AUC) method. Treatment with a non-toxic concentration of DOC did not cause any inhibitory effect compared to the PBS group (Treatment to Control Ratio; TCR: 1.13) (Figure 6A, 6B). Treatment with D7(VL-VH)-PE40 elicited a tumor volume reduction of 42% (TCR: 0.58). Moreover, combination of DOC with the immunotoxin led to a tumor inhibition of 67% (TCR: 0.33) compared to the control. This was calculated to be significant (upper limit of the one-sided Confidence Interval (CI) = 0.63). Compared to the immunotoxin group, combinatorial treatment led to a markedly enhanced growth inhibition of 42% (TCR: 0.58) (Figure 6B). No apparent signs of toxicity were noted during the experiment in all mouse groups.

## DISCUSSION

In this work we have shown that our immunotoxin D7(VL-VH)-PE40 is able to specifically kill PSMA expressing androgen-dependent and –independent prostate cancer cells. A synergistic cytotoxicity was demonstrated with DOC, which resulted in an enhanced antitumor activity *in vivo*.

D7(VL-VH)-PE40 specifically bound to PSMA expressing prostate cancer cells with *Kd* values in the low nM range. This affinity features to be suited for the targeted delivery of cytotoxic agents. ScFv with lower affinity failed to significantly accumulate in tumors, whereas scFv with higher affinities showed a limited tumor penetration [25–27].

D7(VL-VH)-PE40 induced apoptosis in LNCaP and C4-2 cells, which resulted in a high cytotoxicity with IC_50_values in the low picomolar or even femtomolar range. Compared to DOC, the immunotoxin was about 80- to 2000-fold more cytotoxic. This substantial higher efficacy could be based on the different modes of action of both substances. DOC has no enzymatic activity and binds to the β-subunits of microtubulin preferentially, but not solely, of rapidly dividing cells. This results in an inhibition of microtubule dynamics, followed by cell cycle arrest and eventually apoptosis [28]. In contrast, the immunotoxin shows a high and specific binding to PSMA almost exclusively expressed on prostate cancer cells. Moreover, the well-defined intracellular routing of the PE40 domain, which was developed during pathoadaptive evolution of *Pseudomonas aeruginosa*, allows the attack of eEF-2 specifically on the residue diphtamide. Diphtamide is a posttranslationally modified histidine residue, which was exclusively described in eEF-2 [21]. The enzymatic activity of PE40, which enables the ADP-ribosylation of multiple diphtamide residues by only one PE40 molecule, could also contribute to the high cytotoxicity of the immunotoxin [29].

DOC and D7(VL-VH)-PE40 were found to be more cytotoxic against LNCaP than against C4-2 cells. We have chosen these cell lines because they are accepted models representing the androgen-dependent and androgen-independent phenotype of prostate cancer cells [30, 31]. C4-2 cells reflect the CRPC stage in the clinic, which is treated with DOC as a second line therapeutic according to the actual clinical guidelines. C4-2 cells have been shown to be more apoptosis-resistant than LNCaP cells, especially due to an enhanced expression of anti-apoptotic members of the Bcl-2 family [32]. This reflects the clinical situation, where molecular alterations in apoptotic signaling accumulate during androgen deprivation impeding the effective treatment of CRPC [33]. It is therefore conceivable that our immunotoxin could be more effective in hormone-sensitive cancer stages, as was already demonstrated in clinical studies for DOC [34–36].

Furthermore, we were able to demonstrate synergistic effects with DOC and D7(VL-VH)-PE40 in LNCaP and C4-2 cells. *Pseudomonas* Exotoxin A-based immunotoxins are known to induce apoptosis by Bcl-2 associated death promotor (BAD) dephosphorylation and by a degradation of myeloid leukemia cell differentiation protein 1 (MCL-1) [37]. Both molecules decisively contribute to the induction of apoptosis in prostate cancer cells [38]. DOC is able to sensitize prostate cancer cells for apoptosis by activation of p53 or by altering the expression and phosphorylation of members of the Bcl-2 family [39–42]. It is therefore conceivable that DOC could reduce the apoptotic threshold for our immunotoxin by deregulation of pro- and anti-apoptotic proteins. Further experiments are therefore planned to determine the detailed apoptotic mechanisms of DOC and D7(VL-VH)-PE40.

The results of our *in vivo* experiments reflect those of the *in vitro* experiments with higher antitumor activity of combinatorial treatment. We have chosen a treatment schedule with DOC and D7(VL-VH)-PE40 at non-toxic concentrations. With this approach, a single dose DOC did not cause any antitumor activity. However, tumor treatment with the immunotoxin alone led to a tumor inhibition of 42%, whereas pretreatment with a non-toxic DOC concentration increased to an effect up to 67%.

More treatment cycles in a higher number of animals will therefore be included in future experiments to further improve the antitumor activity.

In the past, several *Pseudomonas* Exotoxin A based immunotoxins were generated to target different tumor antigens and were successfully tested in preclinical and clinical trials [43, 44]. With regard to future clinical applications, the expected immunogenicity of D7(VL-VH)-PE40 in humans will be reduced by insertion of a humanized variant of the scFv D7. Moreover, deimmunized PE variants can be incorporated that have also been shown to have a reduced off-target toxicity [45, 46]. Taken together, our anti-PSMA immunotoxin D7(VL-VH)-PE40 is a promising therapeutic agent for a future DOC-based combination therapy of CPRC to improve the therapeutic efficacy and to reduce the adverse side effects.

## MATERIALS AND METHODS

### Cell Lines and reagents

The PSMA expressing, androgen dependent prostate cancer cell line LNCaP, its androgen-independent subline C4-2, and the PSMA-negative cell line DU 145 (ATCC, Manassas, VA, USA) were grown in RPMI 1640 medium (Gibco, Invitrogen, Karlsruhe, Germany) supplemented with penicillin (100 U/ml), streptomycin (100 mg/L) and 10% fetal calf serum (Biochrom, Berlin, Germany) at 37°C in a humidified atmosphere of 5% CO_2_. Docetaxel (DOC) was purchased from Sigma (Taufkirchen, Germany). Authentication of the cell lines was verified by genotyping (CLS Cell Lines Services GmbH, Eppelheim, Germany).

### Cloning, expression and purification of the immunotoxin D7(VL-VH)-PE40

The DNA of the variable domains VL and VH of the anti-PSMA scFv D7 was cloned into the expression vector pHOG21 N-terminally to the cytotoxic domain of *Pseudomonas* aeruginosa, PE40. The immunotoxin D7(VL-VH)-PE40 was periplasmatically expressed in *E.coli* XL-1 blue cells (Agilent Technologies, Santa Clara, CA, USA) and purified using immobilized metal affinity chromatography (IMAC) as described earlier [20]. Purified immunotoxin was dialysed against PBS and sterile-filtered with a 0.2 μm protein filter. Protein content was determined with help of the BCA Protein Reagent Kit (Pierce Technology, Rockford, IL, USA). The immunotoxin was aliquoted and stored at −20°C. The stability of the protein was not affected by a minimum of three repeated freeze/thaw cycles.

### SDS-PAGE and Western Blot

Purification of the immunotoxin preparations was determined via SDS-PAGE according to the manufacturer's instructions (Invitrogen, Carlsbad, CA, USA). For Western-Blot analysis, the immunotoxin was transferred onto nitrocellulose membranes. Membranes were blocked with 5% low-fat milk in PBS-Tween 20 (0.05% v/v) for 1h at RT, incubated with HRP-labeled mouse anti-human c-myc mAb (Roche Diagnostics, Mannheim, Germany) for 1h at room temperature (RT) and developed with 3,3′-diaminobenzidine as substrate. PSMA expression of the cell lines was tested by blotting whole cell lysates (100 μg total protein per lane), followed by PSMA detection (100 kDa under reducing conditions) with the anti-PSMA mAb D20 [19] and rabbit anti-mouse-Ig-POD (Dako, Hamburg, Germany). For the detection of apoptosis 100 μg cell lysates per lane were blotted after immunotoxin or DOC exposure. Membranes were stained with mouse anti-human caspase 3 (ECM Biosciences, Köln, Germany) and rabbit anti-mouse-Ig-POD (Dako) or with rabbit anti human poly(ADP-ribose) polymerase (PARP) (Merck, Darmstadt, Germany) and anti-rabbit-IgG-POD (Sigma). β-actin was detected using a HRP-labeled mouse anti-human β-actin mAb (Merck).

### Flow cytometry

Cell binding of the immunotoxin was evaluated by flow cytometry on PSMA expressing LNCaP and C4-2 cells. PSMA negative DU 145 cells served as control. First, 2 × 10^5^ cells/well in PBS containing 3% fetal bovine serum (FBS) and 0.1% sodium azide were incubated with different concentrations of D7(VL-VH)-PE40 for 1h on ice. After washing with PBS, cells were incubated with mouse anti-human c-myc mAb (Roche Diagnostics) for 40 min on ice. After additional washing, cells were treated with secondary Ab goat anti-mouse Ig-R-PE (Becton Dickinson, Mountain View, CA, USA) in the dark for 30 min on ice. Then cells were washed again and resuspended in PBS containing 3% FBS, 0.1% sodium azide and propidium iodide (2 μg/ml). Mean fluorescence intensities of stained cells were measured and analyzed using a FACScan flow cytometer and the software CellQuest Pro (BD Biosciences, Heidelberg, Germany).

For competitive binding, C4-2 cells were pre-incubated with different concentrations of the anti-PSMA mAb 3/F11 for 1h on ice, followed by the addition of 5 μg/ml D7(VL-VH)-PE40 for additional 1h. The immunotoxin was then detected using rat anti-human c-myc Ab (Invitrogen, Karlsruhe, Germany) and goat anti-rat IgG (H+L)-R-PE (Caltag Lab, Burlingame, CA, USA). Flow cytometric analyses were performed as described above.

### Immunofluorescence

C4-2 cells were grown on glass cover slips (12 reaction fields, Paul Marienfeld, Lauda-Königshofen, Germany) for 24 h. Then the immunotoxin (20 μg/ml) was added and incubated for 4 h at 4°C for binding or at 37°C, 5% CO_2_, for binding and internalization, respectively. For fixation, cells were washed and treated with 2% paraformaldehyde in PBS for 30 min at RT, washed again with 1% bovine serum albumin in PBS, and quenched for 10 min in 50 mM NH_4_Cl in PBS. Mouse anti-human-c-myc mAb (BD Biosciences) and rabbit anti-human EEA1 Ab (Cell Signaling) were added and incubated for 45 min at RT. This was followed by a washing step and incubation with goat anti-mouse IgG-AF488 (Life Technologies, Darmstadt, Germany) and anti-rabbit IgG-DyLight®650 (Abcam, Cambridge, UK) for 45 min at room temperature. Slides were then washed extensively and mounted in Vectashield® containing 4′,6-diamidino-2-phenylindole (DAPI, Vector Laboratories, Inc., CA, USA). Staining was analyzed with help of a Confocal Laser Scanning Microscope (TCS SP2 AOBS, Leica, Wetzlar, Germany; Leica LCS Confocal Software 2.6.1).

### *In vitro* cytotoxicity

Cytotoxicity of the immunotoxin and DOC was measured by WST-1 cell viability assay (Roche Diagnostics). For this, 1.5 × 10^4^ cells were seeded in a 96-well plate and incubated overnight. Then cells were incubated with immunotoxin and DOC alone or in combination. After 24, 48, and 72 h, 15 μl/well WST-1 reagent was added and plates were incubated until the maximum absorbance at 450 nm reached values of about 2.5 optical density (OD). For the determination of the IC_50_-values, defined as the immunotoxin / DOC concentration leading to a reduction of 50% cell viability, non-linear regression [log (inhibitor) vs. response (three parameters)] was estimated (software GraphPad Prism 6). Mutually non-exclusive Combination Index (C. Index) was determined according to Bijnsdorp et al. with values < 0.9 indicating synergistic cytotoxicity of both substances [47].

### *In vivo* experiments

Male SCID mice (5-6 weeks old, 20-25 g) were purchased from Charles River Laboratories (Sulzfeld, Germany) and kept under sterile and standardized environmental conditions. All experiments were carried out according to the animal protection law with permission from the responsible local authorities. For testing toxicity, groups of 3 animals each were treated with single doses of 2, 4, 8, and 16 mg/kg bw DOC i.p. or 0.15, 0.3, 0.6, 1.2, and 2.4 mg/kg bw D7(VLVH)-PE40 i.v. Mice were observed for apparent signs of toxicity (loss of weight and appetite, changes in pelage, fever, tension, apathy, aggression, respiratory disorders, paralyses, death) over a period of 14 days. The maximal dose of each substance, which was tolerated without any signs of toxicity, was defined as the maximal tolerable dose (MTD). After MTD determination of DOC and D7(VL-VH)-PE40, a toxicity experiment was performed treating additional three animals with repeated DOC and immunotoxin doses according to the therapy plan in the next paragraph.

For antitumor therapy, SCID mice were subcutaneously injected with 2.5 × 10^6^ C4-2 cells in 100 μl PBS mixed with 100 μl Matrigel into the right flank (day 1 of treatment). Growing tumors were palpated and tumor diameters were measured in two axes using a vernier caliper. The mean radius (r) was determined to calculate the tumor volume using the formula V=4/3 πr^3^. When tumors reached volumes of about 20 mm^3^ at day 14 of treatment, mice were randomized into four groups. The first group (n = 10) received combinatorial treatment with one dose of DOC (0.5 × MTD, i.p.) at day 15, followed by 3 doses of D7(VL-VH)-PE40 (0.5 MTD each, i.v.) at the days 16, 18 and 20 of treatment. The second group (n = 10) was injected with DOC alone and the third one (n = 10) with D7(VL-VH)-PE40 alone. The fourth group of mice (n = 9) received PBS injections as control. During the experiment, tumor sizes and body weights (bw) of the animals were measured 3 to 4 times a week. At day 29 of the experiment, mice were euthanized. For each animal in each group the time-adjusted Area-Under-the-Curve (AUC) was calculated according to the method described by Wu et al. [48]. To estimate the inhibition effect, the ratio of the means for treatment and control (TCR) was determined. A significant inhibition effect can be claimed, if the upper limit of Fieller's one-sided 95%-confidence interval is below 1 [49].
